# [Retracted] miR‑216b inhibits glioma cell migration and invasion through suppression of FoxM1

**DOI:** 10.3892/or.2023.8663

**Published:** 2023-11-15

**Authors:** Tingting Zhang, Guangtao Ma, Yan Zhang, Hongda Huo, Yuqian Zhao

Oncol Rep 38: 1751–1759, 2017; DOI: 10.3892/or.2017.5824

Following the publication of the above article, a concerned reader reported that certain of the cell invasion assay data shown in Fig. 2D, and the mouse images shown in Fig. 6A, were strikingly similar to data that appeared in the following (subsequently published) article: Li C, Zheng H, Hou W, Bao H, Xiong J, Che W, Gu Y, Sun H and Liang P: Long non-coding RNA linc00645 promotes TGF-β-induced epithelial-mesenchymal transition by regulating miR-205-3p-ZEB1 axis in glioma. Cell Death Dis 10: 717, 2019. The authors regret that the data in question featured in the original *Oncology Reports* article had been re-used, and accept the Editor's decision to retract this article; furthermore, the authors and the Editor apologize to the readership of the Journal for any inconvenience caused.

